# On the importance of integrating comparative anatomy and One Health perspectives in anatomy education

**DOI:** 10.1111/joa.13570

**Published:** 2021-10-24

**Authors:** Sourav Bhattacharjee, D. Ceri Davies, Jane C. Holland, Jonathan M. Holmes, David Kilroy, Imelda M. McGonnell, Alison L. Reynolds

**Affiliations:** ^1^ School of Veterinary Medicine University College Dublin Dublin Ireland; ^2^ Human Anatomy Unit Department of Surgery and Cancer Imperial College London London UK; ^3^ Department of Anatomy and Regenerative Medicine Royal College of Surgeons in Ireland University of Medicine and Health Sciences Dublin Ireland; ^4^ Queens’ College Cambridge University Cambridge UK; ^5^ Department of Comparative Biomedical Sciences Royal Veterinary College London UK; ^6^ Conway Institute of Biomolecular and Biomedical Research University College Dublin Dublin Ireland

**Keywords:** anatomy education, animal models, antimicrobial resistance, biopsychosocial model, comparative anatomy, integration in medical education, One Health, pandemic, zoonoses

## Abstract

As a result of many factors, including climate change, unrestricted population growth, widespread deforestation and intensive agriculture, a new pattern of diseases in humans is emerging. With increasing encroachment by human societies into wild domains, the interfaces between human and animal ecosystems are gradually eroding. Such changes have led to zoonoses, vector‐borne diseases, infectious diseases and, most importantly, the emergence of antimicrobial‐resistant microbial strains as challenges for human health. Now would seem to be an opportune time to revisit old concepts of health and redefine some of these in the light of emerging challenges. The One Health concept addresses some of the demands of modern medical education by providing a holistic approach to explaining diseases that result from a complex set of interactions between humans, environment and animals, rather than just an amalgamation of isolated signs and symptoms. An added advantage is that the scope of One Health concepts has now expanded to include genetic diseases due to advancements in *omics* technology. Inspired by such ideas, a symposium was organised as part of the 19^th^ International Federation of Associations of Anatomists (IFAA) Congress (August 2019) to investigate the scope of One Health concepts and comparative anatomy in contemporary medical education. Speakers with expertise in both human and veterinary anatomy participated in the symposium and provided examples where these two disciplines, which have so far evolved largely independent of each other, can collaborate for mutual benefit. Finally, the speakers identified some key concepts of One Health that should be prioritised and discussed the diverse opportunities available to integrate these priorities into a broader perspective that would attempt to explain and manage diseases within the scopes of human and veterinary medicine.

## Graphical abstract

Acquiring a robust knowledge of comparative anatomy as part of the medical curriculum helps in understanding diseases better. This review article converges the ideas discussed in a symposium on One Health arranged during the IFAA2019 and prioritises an action plan of integrating veterinary knowledge into the medical curriculum.

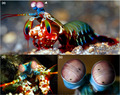

## 1 INTRODUCTION

“Disease is not something personal and special, but only a manifestation of life under modified conditions, operating according to the same laws as apply to the living body at all times, from the first moment until death.” ~Rudolf Virchow (1821–1902).

With an escalation of industrial activity, explosive population growth, increased human mobility, intensification of agriculture, deforestation and climate change, the concept of health is evolving (Rabinowitz et al., 2017). Throughout the advancement of medicine and the philosophy that underscores its core principles, from the Egyptians to the modern world, if one idea has remained constant, it is necessary to understand and define human health from an environmental perspective (Larsen, 2021). Furthermore, the way humans interact with their environment, with its rich and diverse flora and fauna, has also altered over time, and perhaps not always in a beneficial manner (Gibb et al., 2020; White & Razgour, 2020). Widespread deforestation, population increase and concentration in cities have given rise to unprecedented circumstances that pose new healthcare challenges. The occurrence of zoonoses and vector‐borne diseases are perhaps the best examples of how environmental change may give rise to unwelcome events when microbes from animals infect humans, giving rise to diseases that were unknown in the past (Mills et al., 2010). The 47^th^ G7 summit held in the UK during June 2021 identified the One Health approach as a way moving forward and provide sustainable healthcare on a planetary scale. It is also expected to be discussed in the upcoming G20 summit in October 2021.

The changing relationship between humans and the environment has resulted in several viral epidemics and pandemics in recent times, for example, Severe Acute Respiratory Syndrome (SARS), Middle East Respiratory Syndrome (MERS), West Nile virus, Ebola virus and the most recent instance of SARS‐CoV‐2, popularly termed COVID‐19, a pandemic that has disrupted the fabric of normal life on a global scale (Waits et al., 2018; Waugh et al., 2020). The emergence of antimicrobial‐resistant strains across the world has added to the problem. Population growth has intensified both interspecies and intraspecies competition for access to safe water, adequate food and shelter (Reverter et al., 2020). These altered interactions affect disease patterns in humans and their well‐being.

The concept of health should encompass environmental influences and the complex interactions that an individual experiences (Essack, 2018). Having a myopic view of diseases isolated from an individual's environment, including human–animal interactions, will omit important information and obscure a holistic view of disease. Furthermore, clinical research is often conducted using genetically homogeneous experimental animal models and requires expertise to extrapolate the obtained data from animal models to humans. Therefore, modern concepts of health are slowly but steadily embracing the ideas of the *biopsychosocial model*, proposed by the American psychiatrist George Engel (1913–1999), which identifies biological, social and psychological factors as key determinants to explain both well‐being and disease states in humans (Lehman et al., 2017).

## 2 BACKGROUND TO THE SYMPOSIUM

A clinician typically examines a patient in an isolated and controlled environment but must possess the skills to understand that human–environmental interactions may have contributed to the aetiology of a disease. Hence, there is a growing consensus within the community of medical educators that the concept of One Health should be included within the curriculum of human medicine and, if possible, from an early stage (Linder et al., 2020; Wilkes et al., 2018). Such a proposition is underpinned by the fact that there are many ways that human and veterinary medicine professionals can interact, collaborate and develop common knowledge while drawing inspiration from comparative anatomy. However, such a collaboration is rare and one of the primary reasons is that a medical student's packed curriculum leaves little or no room to include One Health concepts (Ribeiro et al., 2019). This lack of integration is particularly apparent in anatomy education, even though applied anatomy provides fertile ground for collaboration between human and veterinary medicine. To stimulate such a collaboration, a symposium was arranged as part of the 19^th^ International Federation of Association of Anatomists (IFAA) Congress in London (UK) from 9 to 11 August 2019. The symposium brought together speakers from human and veterinary anatomy who presented some of their work and perspectives, and stimulated discussion between them and the audience. This report summarises the presentations and discussions, distils the conclusions drawn from the symposium and proposes an action plan.

## 3 FINDINGS AND EXAMPLES

The talks presented in the symposium highlighted some relevant examples of a valuable crossover between human and veterinary medicine, emphasising anatomical knowledge and enhancing the efficacy of current treatment modalities in humans. An interesting example of how knowledge from veterinary medicine can be applied to human medicine relates to the SARS‐CoV‐2 virus. Veterinarians possess extensive knowledge of coronavirus diseases that can be utilised to find a suitable drug to treat SARS‐CoV‐2 patients. An interesting drug candidate could be the nucleoside analogue GS‐441524 that is structurally similar to remdesivir, but less toxic and cheaper (Murphy et al., 2018). After taken up by the cells, it is triphosphorylated and then inhibits viral RNA synthesis. The GS‐441524 nucleoside, when administered subcutaneously in cats (4 mg/kg/day for 12 weeks) suffering from feline infectious peritonitis, a lethal disease caused by feline coronavirus (FCoV) that is closely related to SARS‐CoV‐2, showed excellent efficacy against the FCoV, facilitating full recovery of the feline patients (Pedersen et al., 2019). Such natural animal models of human coronavirus infections could be used in research into treatments for SARS‐CoV‐2 infections, and GS‐441524 may prove superior to remdesivir for the treatment of SARS‐CoV‐2 patients (Yan & Muller, 2020). Such exemplars discuss the pathophysiology of different diseases within a wider One Health context.

### 3.1 Animal models of complex genetic diseases

#### 3.1.1 Canine model for ocular disease

**FIGURE 1:**
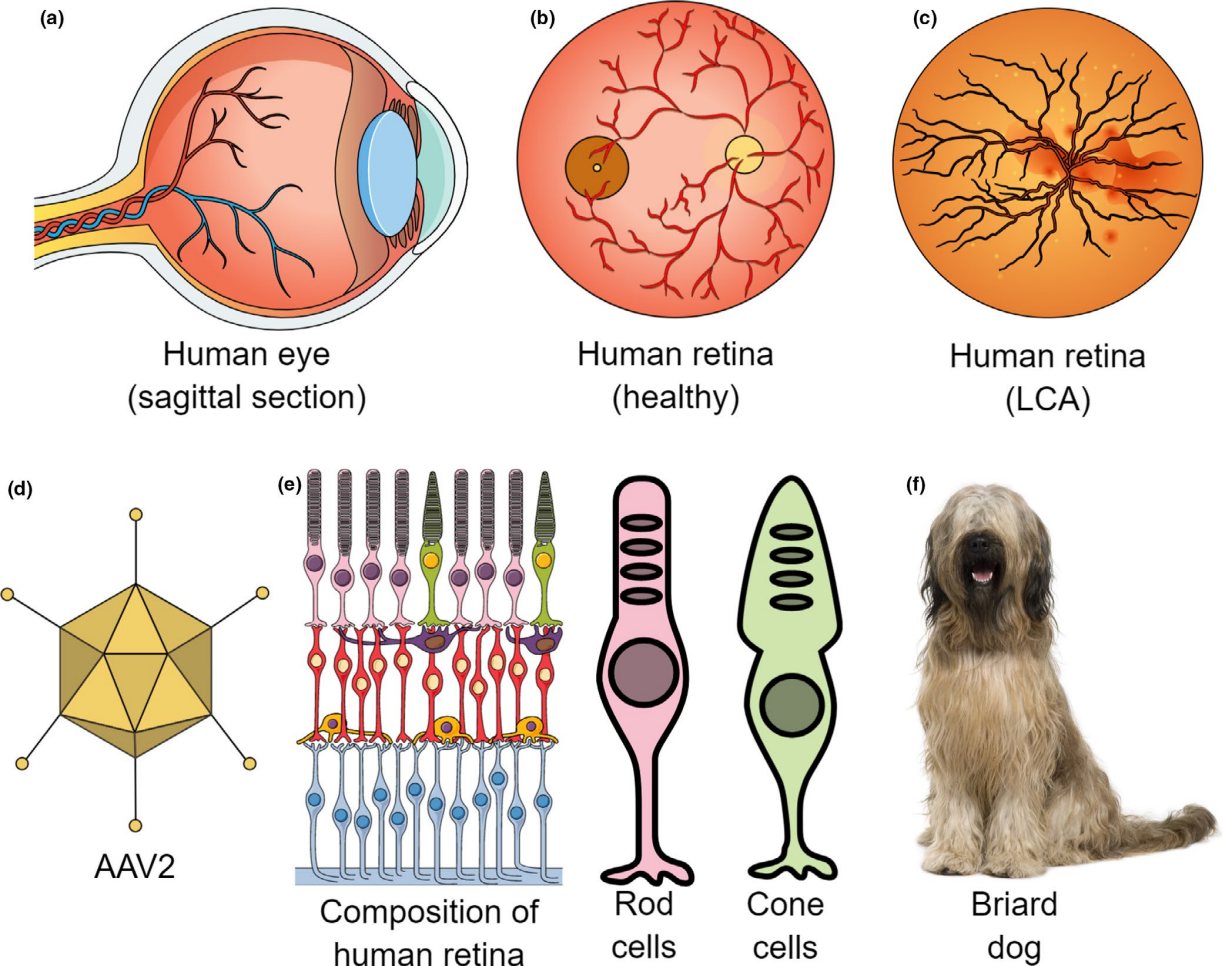
Use of animal models in ocular diseases. Diagrams of (a) sagittal section of a human eye; (b) retina of a healthy human eye; (c) retina in Leber congenital amaurosis (LCA) exhibiting attenuated blood vessels; (d) an AAV2 viral particle used for gene delivery; (e) composition of the human retina with the rod and cone photoreceptor cells; (f) a Briard dog often used in translational studies of Leber congenital amaurosis

Traditionally, a variety of animal models have been used in research into blindness; for example, rodents (e.g., mouse and rat) and larger mammals like rabbits and bovines have been used to investigate posterior and anterior compartment disorders, respectively (Chen et al., 2014). Other animals, such as pigs, have also been used (Penha et al., 2010). However, the eyes of these animals differ from those of humans in attributes including overall ocular morphology, vasculature, distribution of the rods and cones with a preponderance of rod‐based vision to facilitate nocturnal sight. The retina of higher primates and humans contains a *fovea centralis* (Figure 1a,b), a depression on the inner retinal surface of approximately 1.5 mm diameter, packed with cone photoreceptor cells that mediate high visual acuity. In contrast, the canine and feline retina contain an *area centralis* located at the centre of gaze, roughly where the visual axis meets the retina (Rapaport & Stone, 1984), which contains an elevated density of ganglion cells enabling high‐resolution binocular vision. Therefore, extrapolating data derived from animal models to humans requires caution. However, a number of animal models, including Appaloosa horses, dogs, Abyssinian cats, transgenic mice and even cavefish (Amblyopsidae, which have evolved to be completely blind) have been employed as experimental models for research into human blindness (Jeffery, 2001).

The Swiss Briard dog exhibits a congenital form of blindness (Petersen‐Jones & Komáromy, 2014) known as Leber congenital amaurosis (LCA), a rare autosomal recessive disease characterised by retinal degeneration resulting in blindness or severely compromised vision within the first few months of birth (Figure 1c). The incidence of LCA in humans is approximately 1 in 50,000 of the population, and common symptoms include nystagmus, photophobia, strabismus, and rarely, keratoconus (Kumaran et al., 2018). More than 20 genes have been implicated in LCA and this genetic heterogeneity together with the heterogeneous clinical manifestations of the disease make it difficult to treat. However, Briard dogs express a common mutation of the *Rpe65* gene, which has also been shown to cause LCA in humans (Cideciyan, 2010) and thus, can be used as a model of human blindness.

An exciting application of the Briard dog model in human blindness is its use as an experimental platform for investigating gene therapy (Le Meur et al., 2007), involving the injection of wild type *Rpe65* gene encapsulated in a viral vector, such as the adeno‐associated virus type 2 (AAV2), into the eye. AAV2 is a single‐stranded DNA virus without a capsid that can be used for ocular injection of genetic materials in a site‐specific manner (Figure 1d). Using an AAV2‐based vector to deliver genetic materials by subretinal injection has given promising results in restoring vision in the Briard LCA model (Acland et al., 2001), with a single injection demonstrated to reverse the pathology (Wang et al., 2020). Based on this data, this genetic medicinal product went into human clinical trials and *Voretigene neparvovec* (Luxturna^TM^, Spark Therapeutics, Philadelphia, PA, USA) was approved by the US Food and Drug Administration in 2017 as the first *in vivo* gene therapy for treating LCA (Prado et al., 2020). The drug was later also approved by the European Medicines Agency in 2019. It remains an inspiring example of using animal models to develop novel gene‐based therapeutics which could be used to treat inherited disease in humans and animals alike. Further therapeutic enhancements are being investigated with gene‐editing tools like CRISPR/Cas9 (Jo et al., 2019).

#### 3.1.2 Canine models for Chiari malformation

**FIGURE 2:**
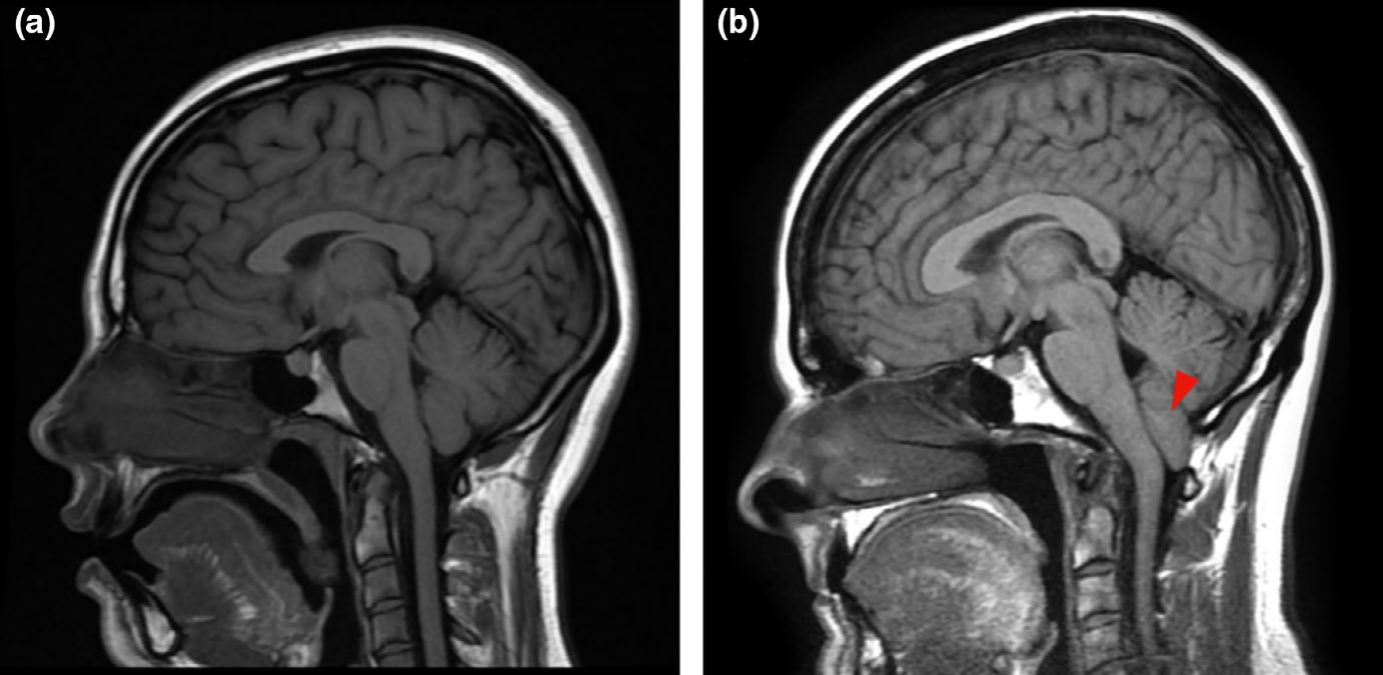
Chiari malformation. The sagittal magnetic resonance imaging (T1) views of (a) normal human brain and (b) Chiari malformation with cerebellar herniation through the foramen magnum into the proximal spinal canal (marked with red arrow). The images are courtesy of Assoc. Prof. Frank Gaillard, Radiopaedia.org (rID: 37605 and rID: 2592)

The Chiari malformation (Hadley, 2002), previously known as Arnold‐Chiari malformation, is a condition in which the caudal part of the brain, including the cerebellum, herniates into the spinal canal (Figure 2). Such a protrusion may be caused by structural defects in the posterior cranial fossa, brain, or spinal cord due to genetic causes, brain tumours, or hydrocephalus. Although such conditions are often asymptomatic in humans, they can create problems due to the exertion of pressure onto the brainstem and spinal cord or obstruction of cerebrospinal fluid flow, often resulting in syringomyelia. Clinical symptoms of such brain herniation include headache, dizziness, paresis, neck pain, and problems with deglutination, vision (e.g., photosensitivity, blurred or double vision) and hearing (e.g., tinnitus and hearing loss). The diagnosis is usually made by magnetic resonance imaging, and unless serious complications arise, such as syringomyelia, the treatment is usually conservative. However, surgical interventions, such as removing bone from the skull base (posterior cranial fossa depression), are required in more serious cases.

Due to the complex aetiology of Chiari malformation, laboratory animal models of this disease have not been generated, hampering understanding of the genetics and pathophysiology underlying this condition. Interestingly, the Cavalier King Charles Spaniel (CKCS) breed of dogs exhibits a very high incidence (95%) of spontaneous Chiari‐like malformation (Olsen et al., 2017) and have a similar pattern of skull development to humans. Therefore, CKCS have emerged as a popular model for investigating the Chiari malformation. Most CKCS develop symptoms, particularly pain and hypersensitivity, which manifest in neck scratching, often unilaterally, and sometimes even while walking. The CKCS model has produced evidence of a developmental anatomical mismatch resulting in a relatively small occipital skull and a large brain in adulthood (Giejda, 2012; Shaw et al., 2012). This size mismatch forces the cerebellum to herniate through the foramen magnum into the spinal canal. Emerging data suggest that due to cerebellar insufficiency and spinal cord compression, affected CKCS exhibit an unstable and abnormal gait (Olsen et al., 2017) and alterations in cerebrospinal fluid composition (Whittaker et al., 2011). Widely believed to be a polygenic trait, the high incidence of Chari‐like malformation in CKCS and other brachycephalic breeds such as Griffon Bruxellois compared with humans has allowed the identification of several candidate genes (Knowler et al., 2018) that will increase understanding of this complex disease and may lead to new therapies for both dogs and humans.

#### 3.1.3 Cancers in humans and animals—a comparative approach

Cancer is the second commonest cause of death worldwide and causes almost nine million deaths annually (Nagai & Kim, 2017). Uncontrolled cell proliferation causing tumour formation, a hallmark of neoplastic cell growth, is often due to gene mutation and occurs throughout the animal kingdom (Albuquerque et al., 2018). While cancer continues to pose challenges for the healthcare sector and exacts its financial burden and toll on human suffering, particularly in the developing world, analysing cancer patterns in the animal kingdom can provide interesting insights.

Faulty cell division holds the key to the initiation of neoplastic growth. Animal bodies are composed of billions to trillions of divisible cells, and the number of cells is dependent on the size and complexity of the animal. It would be expected that larger animals with a greater cell number would be more prone to developing cancer. Surprisingly, an adult human has no greater chance of tumorigenesis than a mouse despite being much larger in size, living longer and most importantly, having many more somatic cells. There are fascinating examples in the animal kingdom of animals much larger than humans, which live longer, and yet exhibit higher resistance towards developing cancer. This paradox is often termed *Peto's paradox*, eponymised after Sir Richard Peto, who suggested that natural selection has allowed larger animals to develop genetic mechanisms to counteract the increased risk of tumorigenesis due to their size (Peto et al., 1975). Bowhead whales can survive for up to two centuries without any incidence of cancer being reported.

Elephants in the wild are known to live for more than 60 years, although their lifespan is typically curtailed in captivity. Such an observation supports the hypothesis that environmental and lifestyle factors associated with exposure to various carcinogens play an important role in developing cancers. Naked mole rats are also known to demonstrate exceptionally high resistance to cancer development and may live for more than 30 years. Perhaps the reason for high resistance against cancer lies in the genetic composition. For example, elephants possess 20 copies of the tumour suppressor gene tumour protein 53 *(TP53)* in their genomes (Abegglen et al., 2015), whereas humans and other mammals usually have only one. Bowhead whales are also known to have a genome that provides resistance against cancer (Keane et al., 2015). Naked‐mole rats have evolved a novel mechanism of cancer resistance. Their fibroblast cells secrete copious amounts of high molecular weight hyaluronan, five times heavier than human or mouse hyaluronan, which accumulates in their tissues (Tian et al., 2013). Low hyaluronidase enzyme activity aggravates hyaluronan deposition, and deposition around fibroblasts provides a safeguard against malignant transformation (Takasugi et al., 2020).

Unfortunately, accurate data about the incidence of different cancers in wild animals is lacking, and data from captive animals may not reflect the situation in wild animals. Therefore, a comparison of cancer incidence trends between humans and animals is difficult, if not impossible. However, some interesting observations can be made. For example, lung cancer, which is the second commonest cancer in humans, is rare in non‐human primates (Giddens & Dillingham, 1971). Similarly, testicular and prostate cancers, which are relatively common in humans, are rare in animals (McClure, 1973). On the other hand, lymphoma has been frequently reported in non‐human apes (Hofmann et al., 2001), but is uncommon in humans, except for adolescents.

Reproductive tract cancers exhibit interesting species variation. For example, uterine adenocarcinoma is relatively common in rabbits (Künzel et al., 2015), but rare in rats. Testicular cancer has been reported in mice, and dogs are known to develop prostate cancer, while the incidence of canine reproductive tract cancer is comparable to humans (Griffin et al., 2018). Reproductive tract cancers are rarely reported in cows, horses and rats but relatively common in seals, with one study reporting a 64% incidence of uterine leiomyomas in Baltic grey seals (Bäcklin et al., 2003). Other genital tract cancers, such as lymphosarcoma and granular cell tumour, also occur in seals (Newman & Smith, 2006). Seminoma and leiomyoma are rare in sea otters (Stetzer et al., 1981), and an incidence of 17.3% in genital tract cancer has been reported in birds (Filippich, 2004).

A detailed discussion of the various cancers reported to occur in animals falls beyond the scope of this review paper. However, some interesting facts are apparent. For example, humans, dogs and cats all exhibit a relatively high incidence of cancer, possibly highlighting the effect of environmental carcinogens and companion animals have proved to be useful in understanding epidemiology, pathophysiology and genetics of common cancers (Schiffman & Breen, 2015). It is possible that the change from a living in a wild habitat to a controlled environment occurred relatively quickly in these animals. Hence, their genetic composition remains mismatched to their current environment and lifestyle, leading to a relatively high cancer incidence. Furthermore, in wild animals, most cancers appear in young adults, while in humans, the overall incidence of cancer increases with age. Perhaps, the relatively great longevity of humans is responsible for the age‐dependent increase in the incidence of cancer. The evolutionary mechanisms that have developed within animals to resist cancer development are diverse and species‐specific. However, there must be strong evolutionary pressure in humans to avoid cancer development before the end of the reproductive phase, but there is no such pressure in post‐reproductive old age, when the incidence of cancer increases. Collectively, potential animal models present an interesting resource for research into cancer and drug development, particularly in cancer types that are more common in animals than humans.

### 3.2 Sex determination in the animal kingdom

Knowledge of veterinary anatomy and physiology is indispensable in understanding some crucial concepts in human medicine. In humans, sex is determined by X and Y chromosomes, with the XY combination in males and XX in females (Snell & Turner, 2018). The presence of the *SRY* gene on the Y chromosome facilitates development of the male sex. In contrast, in birds, a combination of the Z and W chromosome determines sex. However, these Z and W chromosomes neither bear any resemblance to human X and Y chromosomes nor combine like them to determine sex (Smith & Sinclair, 2004). In birds, ZZ determines male and ZW female sex, and no equivalent of the *SRY* gene has been identified. Such findings have baffled scientists; however, recent research has revealed a striking similarity between the genes on human chromosome 9 and the avian Z chromosome. The *DMRT1* gene found on both the Z chromosome and human chromosome 9 might be the sex‐determining gene, given its importance in testicular development (Chue & Smith, 2011). Normal development of testes needs two copies of the *DMRT1* gene, and an absence of one hinders the development of testes resulting in sex reversal in a normal male human (XY) with active *SRY* genes.

The animal kingdom provides us with examples of intriguing social/behavioural factors that can influence sex determination (Casas et al., 2016). For example, clownfish are born male, and their clusters maintain a strict *dominance hierarchy*, in which only one female fish, usually the largest and most aggressive fish, is allowed per cluster. The female fish mates with the fully grown and sexually mature male fish in the cluster *via* external fertilisation to produce offspring that are all males at birth. However, if the female fish is removed from the cluster, for example, due to death, then the surviving dominant male transforms into a female while one of the juvenile males develops into a sexually mature male fish. The mechanism of such *sequential hermaphroditism* in clownfish is still not well understood (Todd et al., 2016). However, the sexually mature male fish within the cluster possess testes containing undifferentiated cells that can be precursors of ovaries. The female fish in the cluster may secrete chemical(s) that inhibits the latent ovarian cells from growing. If the female fish is removed from the cluster, an absence of these chemicals may trigger degeneration of testes and the development of precursor cells into ovaries.

**FIGURE 3:**
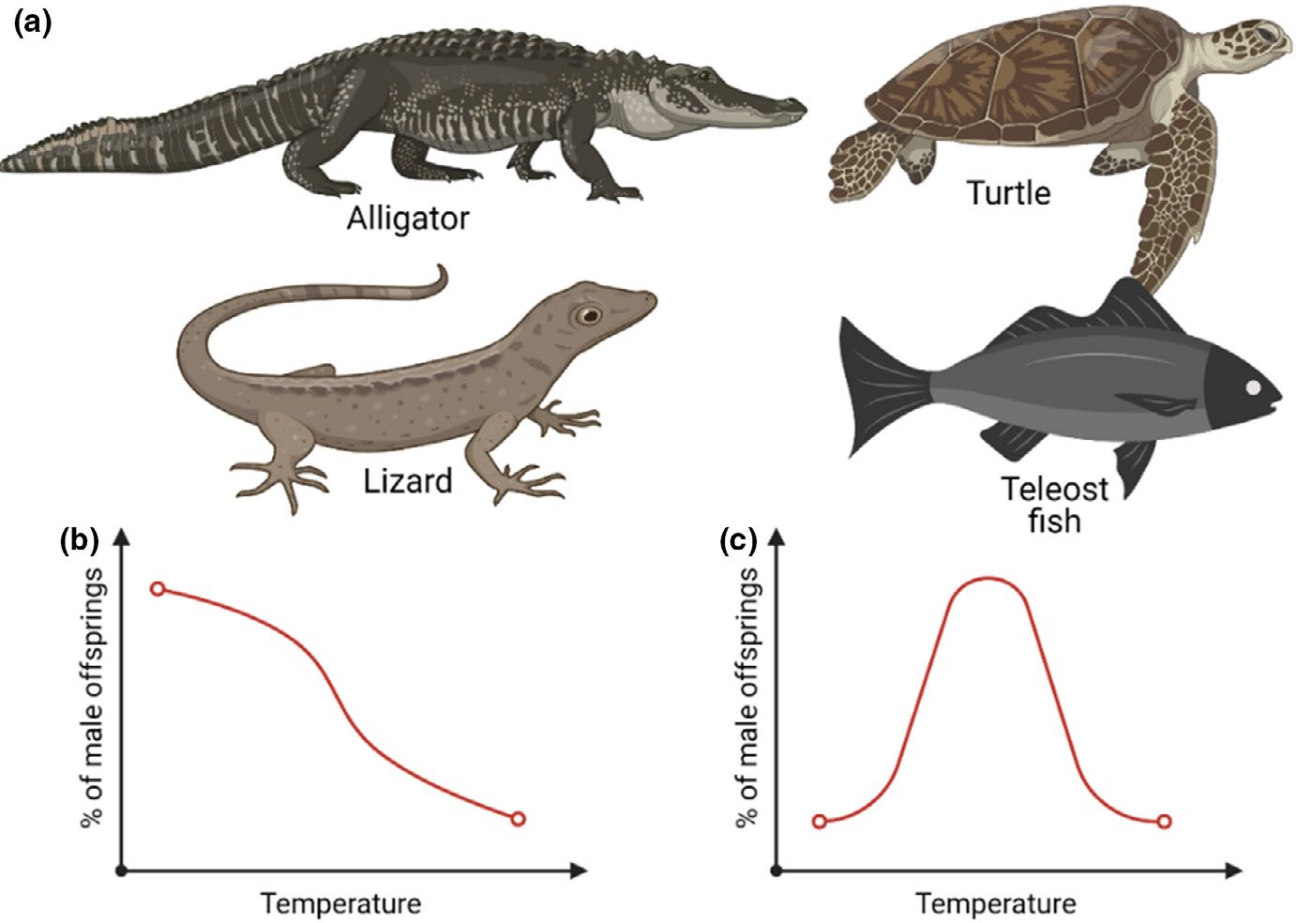
Temperature‐dependent sex determination (TSD) in reptiles and teleost fishes. (a) Some animals demonstrating TSD; patterns of TSD with one (b) and two (c) transitional zone(s)

Environmental factors can influence the process of sex determination; for example, temperature has been shown to determine sex after fertilisation (Crews, 1996). Such temperature‐dependent sex determination (TSD) occurs in reptiles (e.g., turtles, crocodiles and alligators) and teleost fishes (Figure 3a). For example, in turtles, if the incubation temperature of the eggs is below ~82°F (~28°C), then the offspring are mostly male, while if it is higher, they are mostly female (Bull, 1980). In contrast, in tuatara *(Sphenodon punctatus)*, an endangered reptilian species native to New Zealand, mostly females are born below the transition temperature of ~70°F (~21°C) and predominantly males above it (Figure 3b). The TSD in some lizards and crocodiles may even demonstrate two transition zones (Figure 3c; González et al., 2019). Interestingly, the variation in transition temperature for sex determination can be as little as 1–2°C, illustrating the considerable temperature sensitivity of the process. TSD typically depends on a *thermosensitive period*, usually in the middle of the incubation period, when sex is determined. Once the thermosensitive period has passed, sex cannot be changed.

The mechanism(s) of TSD is/are unclear, although there may be proteins whose activities are switched on or off at above or below the transition temperature. However, emerging data suggest that temperature‐dependent activation of endocrine systems, particularly oestrogen, may have a role. The synthesis of oestrogen is highly dependent on the enzyme *aromatase*, a monooxygenase enzyme that is part of the cytochrome P450 (CYP450) superfamily (CYP19A1), and its activity is highly temperature sensitive (Yamaguchi & Iwasa, 2018). Cortisol has also been proposed as to be involved in TSD (Yamaguchi et al., 2010). Due to climate change and gradually increasing temperature, some animals change their behaviour to maintain optimal sex ratios. For example, it has been noted that some animals have begun to prepone their reproductive cycles, and there is concern that with increasing environmental stress, some species will ultimately fail to continue to adapt and, hence, face extinction.

Climate change and global warming may be poised to influence human fertility, possibly negatively (Fisch et al., 2003). However, the scientific community remains unsure of what the impact of such changes on human fertility will be. Unabated greenhouse gas emission is estimated to raise global average temperature by 5°C until 2100 AD (Tollefson, 2020) with the melting of the polar ice caps resulting in rising sea levels that risk submerging multiple landmasses (Wunderling et al., 2020). A high ambient temperature discourages physically strenuous exercise like copulation and can be detrimental to the health of infants and the elderly (Smith, 2019). A study conducted in the US in 2015 demonstrated that the birth rate in Louisiana decreased by 0.4% on hot days with a temperature of ≥32°C, while the normal average total birth rate was retained once the daily temperature returned to normal (27°C) (Barreca et al., 2018). There is currently very little or no data available on the impact of high temperatures on human fertility in the developing parts of the world, where most of the global population lives in a climate that is most at risk of rising temperatures. The impact of climate change and global warming also threaten to increase global inequality and alter psychosocial behaviour, with significant implications for human society (Casey et al., 2019).

### 3.3 Photochemistry of respiratory pigments

**FIGURE 4:**
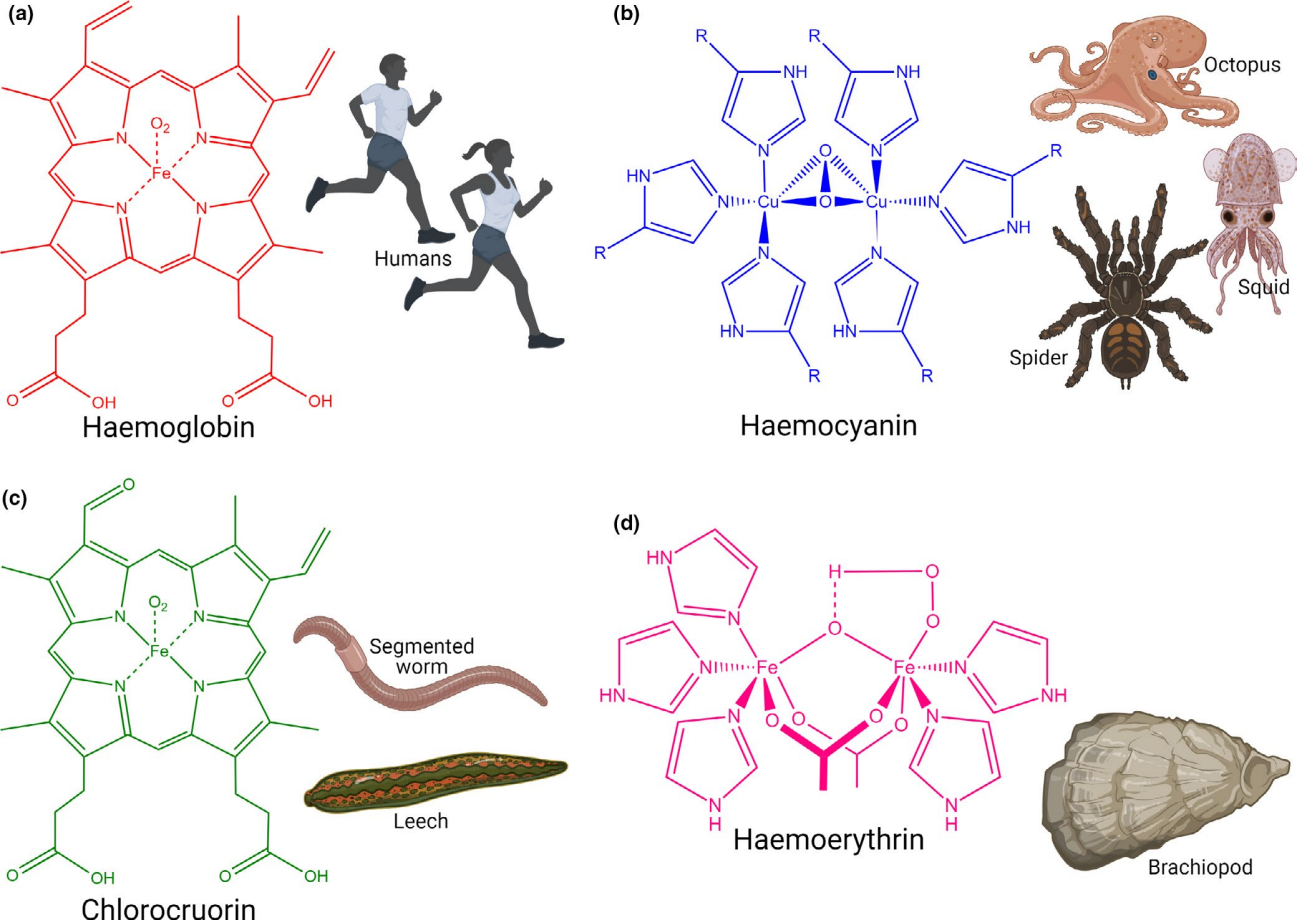
The different respiratory pigments occurring in the animal kingdom. (a) haemoglobin, (b) haemocyanin, (c) chlorocruorin and (d) haemoerythrin possess unique molecular structures in oxygenated forms as shown here and, thus, impart different colours to the blood

There is considerable variation in the colour of blood within the animal kingdom (Figure 4). While red is the commonest colour in vertebrates due to the presence of haemoglobin (Hb), blue (squid, octopus, spiders, crustaceans), green (leeches and segmented worms) and violet (marine worms and brachiopods) coloured blood also occur (Figure 4a). These colour variations are due to the characteristic molecular structure of the respiratory pigments present in the blood (Terwilliger, 1998). Haemocyanin, chlorocruorin and haemoerythrin are responsible for the blue, green and violet blood colours, respectively. In contrast to Hb, haemocyanin (Figure 4b) contains copper, and rather than being bound to the red blood corpuscles, it floats freely in the blood. If oxygenated, haemocyanin is blue, but colourless in the deoxygenated state. Chlorocruorin pigment is similar in molecular structure to Hb and carries iron (Figure 4c), although it does not contain any chlorine as its name might suggest. It appears green or light green in oxygenated and deoxygenated states, respectively. Finally, hemoerythrin, another iron‐bearing respiratory pigment (Figure 4d), has only a quarter of the oxygen‐carrying capacity of Hb. It is violet in an oxygenated state and almost colourless when deoxygenated (Ascenzi et al., 2007). These respiratory pigments are crucial in carrying oxygen to the tissues. Any failure in such oxygen‐carrying capacity would result in hypoxemia, as typically occurs in patients suffering from acute respiratory distress syndrome, for example, in SARS‐CoV‐2 patients, and requires external oxygen supplementation with a target SpO_2_ of 92–96% (Shenoy et al., 2020).

**FIGURE 5:**
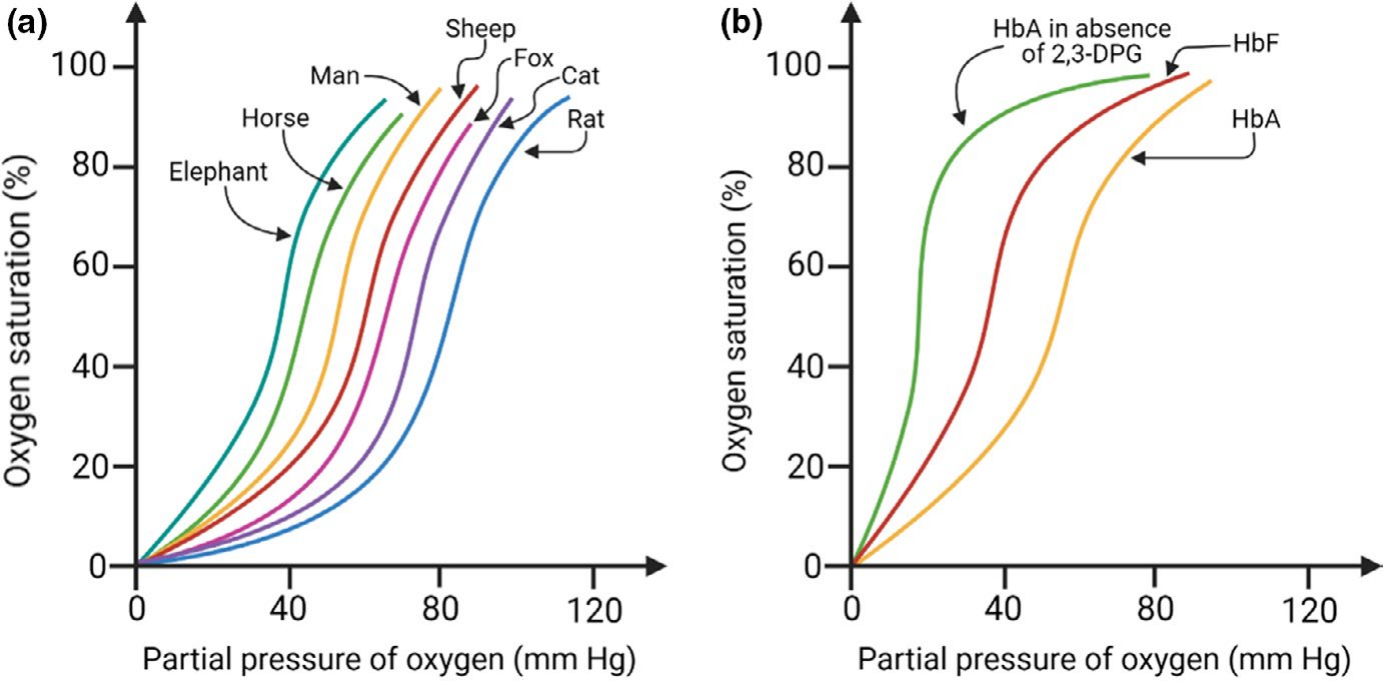
The oxygen dissociation curve of haemoglobin. (a) A right shift of the curve occurs in mammals with decreasing body size; (b) a left shift of the curve occurs in foetal haemoglobin (HbF) or native adult haemoglobin (HbA) without 2,3‐DPG compared with HbA

The oxygen binding and dissociation properties of adult Hb (HbA) demonstrate interesting species variation. For example, an increase in partial environmental pressure of oxygen (pO_2_) causes a right shift of the oxygen dissociation curve. Thus, with the transition from gills to lungs as the major respiratory organ, a right shift of the oxygen dissociation curve is observed since pO_2_ in the air is higher than water. A right shift of the dissociation curve also signifies increased oxygen release from oxygenated Hb, for example, during exercise. A similar trend is noted with evolution from larger to smaller mammals (Figure 5a). Such a size‐dependent right shift of the dissociation curve in mammals arises due to the increased metabolic rate per unit mass in smaller species, which demands higher oxygen consumption (Storz, 2007). A right shift of the dissociation curve ensures more oxygen is released from its bound state in Hb and satisfies the increased tissue oxygen demand. Conversely, a left shift of the dissociation curve occurs in foetal Hb (HbF) and in 2,3‐diphosphoglyceric acid (DPG), characterised by a higher affinity towards oxygen compared to Hb (Figure 5b; Thomas & Lumb, 2012).

### 3.4 Comparison between human and avian respiratory systems

**FIGURE 6:**
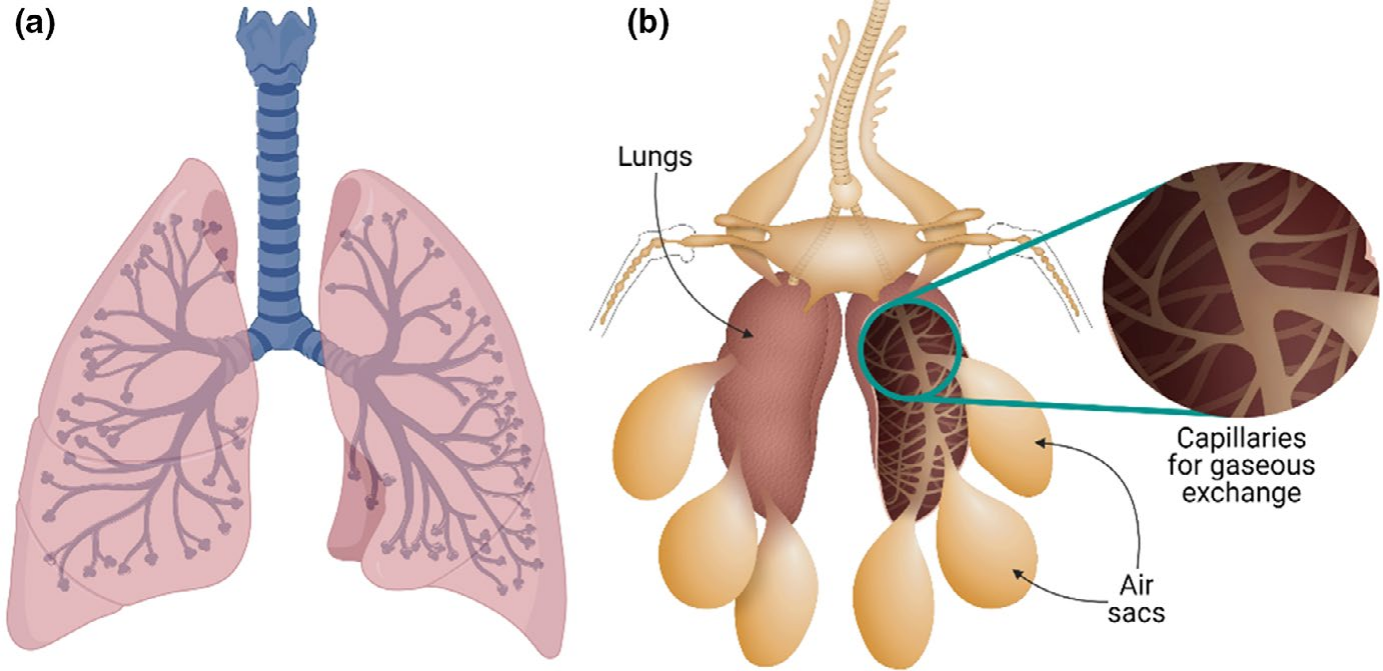
The avian respiratory system. Diagrammatical representations of (a) human respiratory system for comparison; (b) avian respiratory system in which a series of air sacs are connected to the lungs to maintain a unidirectional airflow, the inset shows the series of capillaries in a bronchus for gas exchange

Unlike mammals, including humans (Figure 6a), the avian respiratory system is characterised by unidirectional airflow in which expiration, and not inspiration, is the active process (Brown et al., 1997). Avian lungs are small and only contribute ~2% of their body volume but in addition, birds carry 7–9 air sacs connected to their lungs (Figure 6b). Interestingly, these air sacs do not contribute to gaseous exchange, but help complete a circuit for unidirectional airflow through the respiratory system. These air sacs extend into bones, including the humerus, femur, vertebrae and skull bones. In contrast to mammals, birds lack a thoracic diaphragm and as a result, an infection in the air sacs due to inhalation of contaminated air can quickly spread into the abdominal cavity and bones. The gaseous exchange in mammalian lungs occurs through the walls of microscopic air sacs (alveoli), whereas in birds, such exchange happens through the thin walls of air capillaries (Figure 6b).

Avian respiration consists of four stages: inspiration, expiration, inspiration and expiration (Heard, 1997). During the first inspiration, air flows *via* the trachea to the caudal air sacs, then the first expiration enables the air in the caudal air sacs to move into the ventral and dorsal parabronchi of the lungs. The parabronchi divide inside the lungs to form capillaries with walls only one to two cells thick, facilitating gaseous exchange by diffusion. The second inspiration displaces air from the lungs into the cranial air sacs, followed by the second expiration, which channels the air in the cranial air sacs to the exterior *via* the syrinx, trachea, larynx, nasal cavity and nostrils. The avian respiratory system enables the passage of a greater volume of fresh air and is more efficient than the mammalian respiratory system in providing oxygen while breathing. However, such enhanced ventilation also makes birds more prone to toxic vapours than mammals (Fedde, 1998).

The mammalian respiratory system is markedly different from the avian one that is biomechanically superior and more efficient. Unlike the mammalian respiratory system, the avian respiratory system has managed to keep its two pivotal functions, exchange of gas and ventilation, separate; while the lungs conduct gaseous exchange, the air sacs control ventilation (West et al., 2007). For unclear evolutionary reasons, the mammalian respiratory system has combined both these functions in the lungs. Moreover, the mammalian respiratory tract must be flexible to accommodate the inflation and deflation caused by inspiration and expiration, whereas the avian respiratory tract is much more rigid. The expansion due to inspiration may cause bleeding in human alveoli that have capillaries with thin walls (thickness 0.2–0.3 µm), as has been reported to occur in athletes after strenuous exercise (Hopkins et al., 1997). Such bleeding cannot occur in avian air sacs that lack the capillaries that characterise human alveoli. The reciprocating ventilation in the mammalian respiratory system also results in a large residual gas volume (~3 L) after each expiration. As a result, the inspired 0.5 L of air must mix with the residual gas and can only reach the peripheral regions of the lungs by a combination of convection and diffusion. Hence, peripheral alveoli may receive less oxygen than central alveoli, especially under physical stress, which is not the case in birds. The human respiratory system also suffers from the disadvantage that in localised airway obstruction due to aspiration in post‐operative patients, asthma, or chronic bronchitis, gas exchange is impaired (Miskovic & Lumb, 2017). In contrast, in birds, even if such obstruction of airflow occurs, gas exchange continues in the pulmonary alveoli while the inhaled aspirate is deposited in the avascular air sacs. Furthermore, the continuous inflation and deflation of the lungs in the mammalian respiratory system contributes to a higher incidence of emphysema (West et al., 2007).

### 3.5 Eyes of mantis shrimp

**FIGURE 7:**
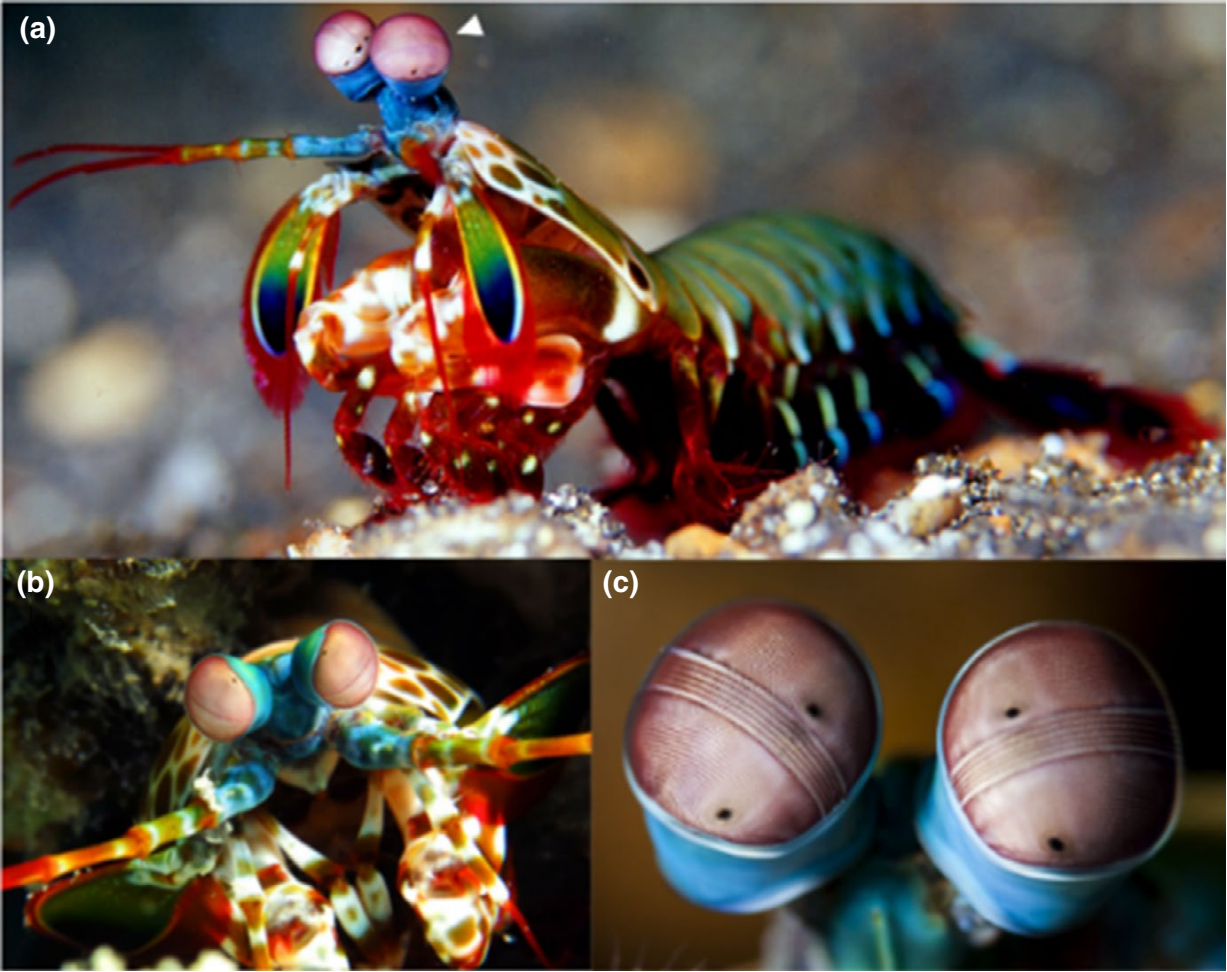
The powerful eyes of mantis shrimp. (a) the mantis shrimp with its two eyes (indicated by the white arrowhead, which are mounted on antennas to augment its field of vision); (b) anterior view of the two eyes of mantis shrimp; (c) higher magnification views of the eyes of mantis shrimp showing the arrangement of the photoreceptors that detect polarised light in the middle band

The eyes of humans and higher order primates are mostly equipped with three types of colour receptors (red, green and blue), although most other mammals are dichromatic. Birds have four types of colour receptors (red, green, blue and ultraviolet), and some invertebrates demonstrate more powerful colour perception than vertebrates; for example, butterflies have five different photoreceptors that enable them to detect ultraviolet light, the usual red‐green‐blue triad and to facilitate an enhanced resolution between colours (Gerl & Morris, 2008). However, none of the above can compete with the mantis shrimp, a marine crustacean with the most complex eyes in the entire animal kingdom, with 16 different colour receptors (Figure 7a). Such incredible vision in mantis shrimp is often attributed to the diverse expression of the opsin proteins due to specific mRNA expression that leads to a G_q_‐mediated phototransduction pathway (Donohue et al., 2017).

The compound eyes of mantis shrimp are composed of many thousands of ommatidia, and can differentiate spectra within colours, and detect polarised light (Figure 7b). The ommatidia are arranged in bands, with most photoreceptors being arranged in six rows in the middle band (Donohue et al., 2018). The top four rows comprise 12 different photoreceptors that detect colours, and at least four of these detect ultraviolet light (Figure 7c), while the fifth and sixth detect circularly and linearly polarised light. The ability to detect polarised light, particularly in the horizontal, vertical and two diagonal directions, is also present in other invertebrates, such as octopus, squid, crab and cuttlefish. However, the mantis shrimp is the only animal known to detect circularly polarised light moving in both clockwise and anticlockwise directions (Daly et al., 2016). The ability to detect polarised light is helpful in a marine environment where sunlight regularly polarises after reflecting from seawater (Kleinlogel & White, 2008). Mantis shrimp can move their two eyes independently, and all three parts of each eye can focus on a narrow strip of an object.

Additionally, their eyes are mounted on antenna‐like structures, which, coupled with the wide range of eye movements, can provide the animal with great visual precision and the ability to scan its environment. It has been suggested that the eyes of mantis shrimp are used for visual signalling, including during mating (Cronin et al., 2003). Male mantis shrimp are known to perform aggressive dancing to attract females and, at the same time, deter male competitors. During such dancing, the high reflectance of coloured patches on their bodies appears attractive to females. The highly sensitive eyes of mantis shrimp may be crucial for detecting such visual cues, and it has also been claimed that the highly social mantis shrimp utilise their ability to detect circularly polarised light for communication within their social groups (Gagnon et al., 2015). The unique capability of mantis shrimp to detect polarised light has inspired researchers developing high‐resolution cameras for underwater imaging (Garcia et al., 2017) and detection of cancer (York et al., 2014). Moreover, the mantis shrimp presents an excellent model for teaching ocular anatomy and physiology, which is influenced by multiple factors, including environmental (e.g., extreme temperature, pollutants, ultraviolet radiation, chemicals, infection, iatrogenicity, toxic fumes; Johnson, 2004) and genetic ones (Sheffield & Stone, 2011).

### 3.6 Anatomy of the equine auditory (Eustachian) tube

The equine Eustachian tube possesses a diverticulum, known as the *guttural pouch*, a space for the accumulation of air (350–400 ml), and in pathologic states, pus (Baptiste, 1998). It is a typical anatomical feature of the odd‐toed ungulates, including zebras, donkeys and mules. These pouches are present bilaterally caudodorsal to the pharynx, ventral to the wings of the atlas (C1) vertebra and medial to the stylohyoid bone while being connected by the auditory tube to the nasopharynx. The guttural pouch's relations include the salivary glands (parotid and mandibular) and the pterygoid muscles. The projection of the stylohyoid bone divides each pouch into medial and lateral compartments. The pouches are lined by pseudostratified columnar epithelium, lymph nodes and mucus‐secreting goblet cells; and are situated in vicinity of the glossopharyngeal, vagus, accessory and hypoglossal cranial nerves, the sympathetic trunk from the superior/cranial cervical ganglion and both the external and internal carotid arteries.

**FIGURE 8:**
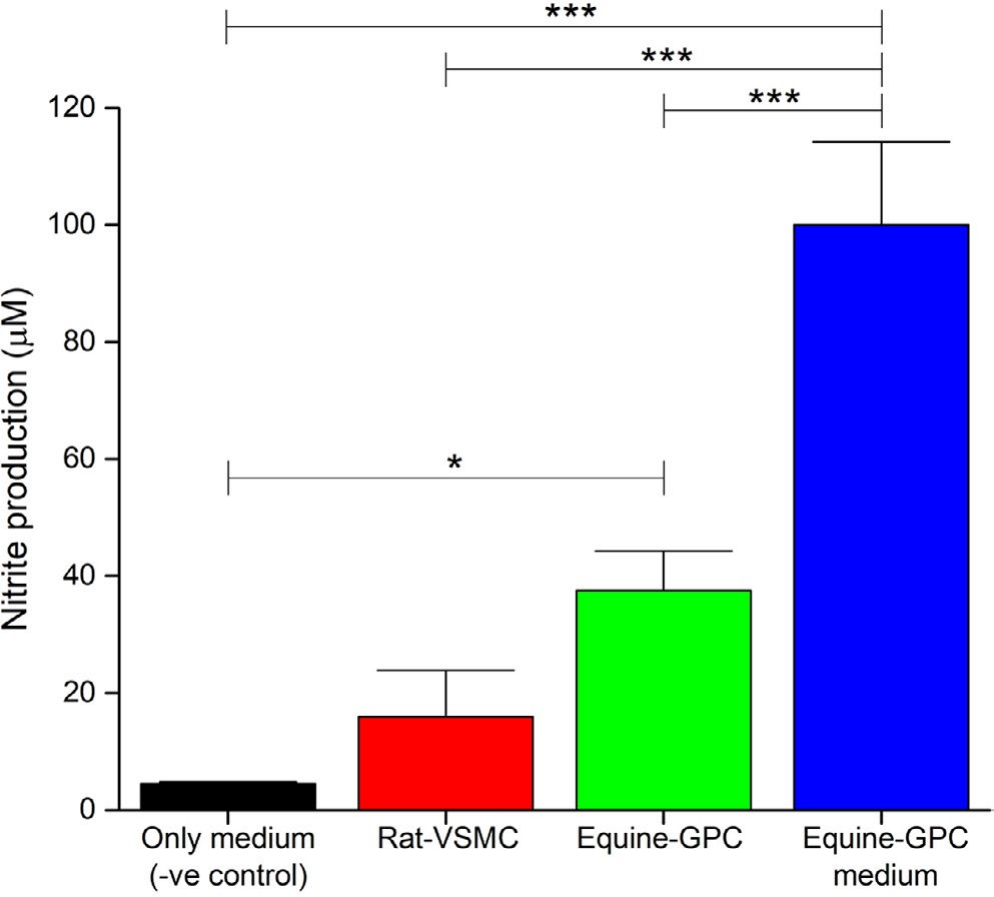
The role of equine guttural pouch cells in secreting vasoactive substances. A bar diagram showing nitrite production by rat vascular smooth muscle cells (Rat‐VSMCs), homogenised equine guttural pouch cells (Equine‐GPCs), and medium in which equine guttural pouch cells had been cultured (Equine‐GPC medium), with cell culture medium only as a negative control (only medium ‐ve control). Results are shown as mean ± standard deviation (*n* = 3). Data are expressed as % of Equine‐GPC medium. Significant differences of *p* < 0.05 and *p *< 0.01 identified in data points, when compared with the negative control, are marked with an asterisk (*) and triple asterisk (***) symbols, respectively

The function of the guttural pouch remains unclear. Some argue that such an air‐filled space allows the blood passing through the carotid vessels to cool down by convection and, thus, protects the brain from hyperthermia, particularly during exercise (Baptiste et al., 2000). It has also been speculated that these pouches assist in pressure regulation during exercise as horses are obligate nasal breathers. Recently, a group in the University College Dublin School of Veterinary Medicine investigated the role of guttural pouch in secreting vasoactive substances by analysing the following for nitrite content: (i) cell culture medium in which rat vascular smooth muscle cells (rat‐VSMCs) were incubated for over 72 h, (ii) homogenate derived from equine guttural pouch cells (equine‐GPC) and (iii) cell culture media in which equine guttural pouch cells were cultured (equine‐GPC medium) for 72 h. A cell culture medium without cells was used as a negative control. The guttural pouch homogenate contained a significantly higher (*p* < 0.05) nitrite content than the negative control and the culture medium in which the rat‐VSMCs were cultured, whereas the culture medium in which equine‐GPCs were cultured demonstrated an even higher (*p *< 0.01) nitrite content than the three other substances investigated (Figure 8, unpublished data). These data indicate a role for the guttural pouch in producing vasoactive substances that may help maintain blood pressure during stressful states, including exercise.

The guttural pouch is notorious for infection that can cause an accumulation of pus within it. Such empyema can cause swelling behind the mandible, and the commonest microorganism to cause a mucopurulent empyema is *Streptococcus equi* (Boyle et al., 2017). Although less common than bacterial infections, fungal infections (Eichentopf et al., 2013), such as Aspergillosis, can cause life‐threatening pathology (Pollock, 2007). Such infection often follows a guttural pouch tympany where a large amount of air is trapped within the pouch leading to infection. Arabian horses are particularly susceptible to such tympany. Infection in the guttural pouch can cause severe haemorrhage (often noticed as bilateral epistaxis) and compromised function of the CN IX–XII, because the pouch is closely related to major vessels and cranial nerves (Hardy & Léveillé, 2003). Usual diagnostic modalities include radiographic as well as endoscopic examination. Biochemical analysis of the lavage fluid after washing the pouch with saline can provide useful information. In milder cases, the application of antimicrobials with recurrent lavage and drain insertion usually remedies the infection. However, more serious cases require surgical intervention.

Despite the absence of guttural pouches, middle ear infections, commonly termed *otitis media*, are common in humans (Schilder et al., 2016). However, instead of fungi, bacteria like *Streptococcus pneumoniae*, *Moraxella catarrhalis* and *Haemophilus influenzae* are the main causative agents of such infections (Perez et al., 2014). These microbes typically gain access into the middle ear through the Eustachian tube from the respiratory tract and often, like horses, cause accumulation of pus (empyema) in association with earache, fever, impaired hearing, general fatigue and drainage in case a rupture of the eardrum (Worrall, 2007). As in horses, antibiotic therapy is often recommended in treating such middle ear infections (Suzuki et al., 2020). However, unlike horses, the absence of guttural pouches in man ensures that erosion of cranial nerves and major vessels causing neuronal insufficiency and profuse haemorrhage are usually avoided. Therefore, comparing equine guttural pouches and their infections with human otitis media can provide a wider perspective on the anatomy, function and clinical importance of the pharyngotympanic tube.

### 3.7 Nervous system of an octopus

The Octopus, the only cephalopod without a shell, possesses a unique nervous system and is perhaps the only invertebrate that comes close to humans in intelligence. Information about octopus evolution remains sparse because few soft body parts remain in the fossil record. However, it is generally accepted that the vertebrate and mollusc lines separated almost 550 million years ago from their common ancestor (Wanninger & Wollesen, 2019). Currently, more than 300 species of octopus have been identified, which live mostly in the marine environment and vary considerably in size and weight. The octopus nervous system comprises a comparable number of neurons (~500 billion) to mammals like dogs (Shigeno et al., 2018). However, the anatomy of the octopus’ central nervous system is very different from that of vertebrates. Most of its neurons are arranged in its arms, receiving tactile sensation, olfactory and gustatory information (Zullo et al., 2019). Although slow learners, under experimental conditions, octopuses can be trained to navigate a maze and unscrew the lid of a jar to gain a reward. There is evidence that octopuses may also participate in simple games. Additionally, despite not being a social animal, captive octopuses have been demonstrated to recognise and interact with their human keepers and to be able to attempt to leave their aquarium incognito (Amodio et al., 2019).

Like other cephalopods, the octopus has a ladder‐like nervous system arranged in pairs of connected ganglia, some of these ganglia are combined at the front of the body to form the brain. The arms of an octopus harbour almost twice as many neurons as its brain, with each sucker containing at least 10,000 neurons. When an octopus holds an object with the terminal part of its arm, two counter directional muscle contraction wavefronts arise, one spreading from the base of the arm towards the tip and the other from the tip towards the base. Where these wavefronts meet, a *temporary elbow* is formed (Hochner, 2012). Initially, it was thought that the brain and nerve centres in the arms were not connected, but at least in some species, the octopus can extend an arm towards an unknown object or food that is out of its reach, indicating a brain‐driven action that may be refined by visual input. The eyes of an octopus are like those of vertebrates in that they have a lens to allow focussing on an object. Like vertebrates, octopuses also sleep, but it is unknown whether they experience dreams or rapid eye movement sleep (Keene & Duboue, 2018).

The octopus nervous system provides research opportunities for neuroscientists and evolutionary biologists. As mentioned earlier, the invertebrate ancestors of octopus diverged from the lineage of vertebrates around 550 million years ago, when the common ancestor probably had a simple morphology with an uncomplicated nervous system where the number of neurons was probably in thousands. From the time of divergence, the evolution of cephalopods has followed a very different trajectory from that of vertebrates, so that anatomically, extant octopuses and humans have very little in common (Schnell et al., 2021).

Despite having a very different nervous system from that of vertebrates, octopuses have developed consciousness, reasonable cognitive ability, memory and sleep with some indication of rapid eye movement during sleep (Shigeno et al., 2018). Additionally, they have developed analogous centres to parts of vertebrate brains. For example, the cerebral cord, dorsal basal lobe, anterior basal lobe, optic lobe and peduncle lobe in an octopus are functionally equivalent to fore‐ and midbrain, thalamus, basal ganglia, tectum and cerebellum in the vertebrates, respectively. Similarly, the pedal and palliovisceral cords in octopuses are analogous to vertebrate hindbrain and spinal cord (Bullock, 1965; Butler & Hodos, 2005; Hartenstein, 2006).

In addition, to compensate for the lack of white matter in the vertebrate central nervous system, which facilitates rapid transmission of electric signals, octopuses have evolved giant neurons with axons almost 1 mm in diameter (Williamson & Chrachri, 2004). The octopus nervous system presents a model in which similar cognitive and behavioural capabilities to those of vertebrates have emerged *via* a parallel evolutionary route, albeit with profoundly different anatomy. Understanding the nervous system of octopus may provide us with further insights into the neural basis of behaviour and the less understood mechanisms of our nervous systems with pharmacotherapeutic implications for a variety of neuronal disorders. It is worth mentioning here that the Nobel Prize‐winning seminal research work in 1963 by Alan Hodgkin (1914–1998) and Andrew Huxley (1917–2012) on the ionic mechanism of propagation of nerve impulse was conducted on the giant neurons of another cephalopod longfin inshore squid (*Doryteuthis pealeii*).

### 3.8 Anatomy of the shoulder joint

**FIGURE 9:**
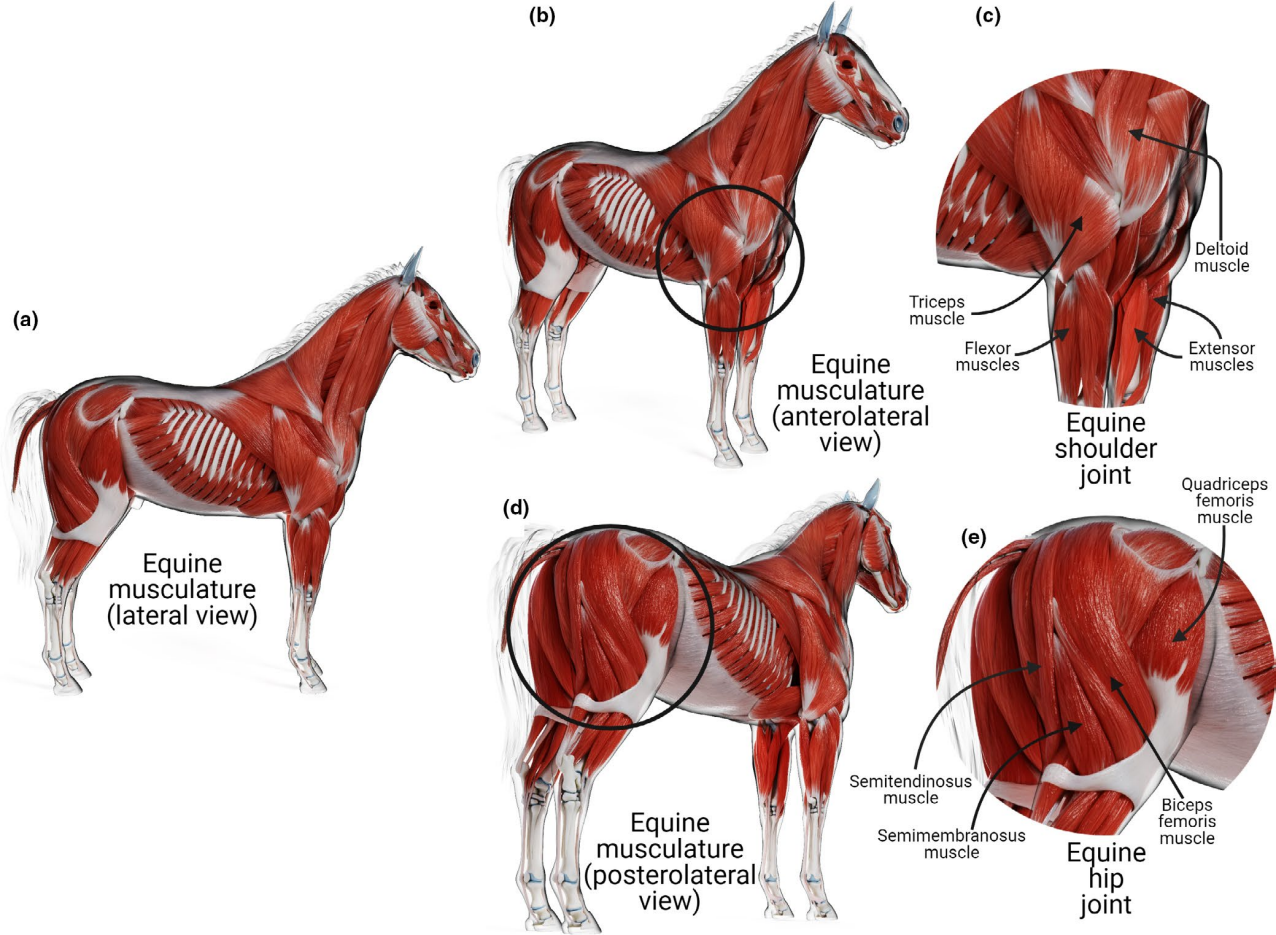
Equine musculature. Diagrammatic representations of (a) lateral view; (b) anterolateral with a close view of the equine shoulder (c) joint marked with a circle; (d) posterolateral view of equine musculature with a close view of equine hip (e) joint marked with a circle

The limbs of many terrestrial mammals have evolved to facilitate (fast) running. Some of these cursorial adaptations include the development of a lighter distal limb *via* a variety of mechanisms, including loss of multiple bones, elongation of the distal limb and restriction of limb joint (e.g., the shoulder joint) movement, mostly to the sagittal plane (Hudson et al., 2011; Payne et al., 2005; Stein & Casinos, 1997). The clavicle no longer exists in most domestic animals. Cats possess a vestigial collar bone, while in dogs, a fibrous clavicular ligament is present (Nagashima et al., 2016; de Souza Junior et al., 2020; Voisin, 2006). Thus, a skeletal bridge between the axial skeleton and forelimbs has been lost. The connection is maintained by a *synsarcosis* of muscles, forming a *trunco*‐*brachial* junction between the forelimb and the trunk. Thus, in these animals, the entire torso hangs from its forelimbs with the help of the *serratus ventralis* muscle (Arias‐Martorell, 2019). In a fast and heavy animal like a horse, the muscles are rich in fibrous tissue to provide robustness and maintain the connectivity between the axial skeleton and limb (Figure 9; Skalec & Egerbacher, 2017). Additionally, the pectoral muscles ventrally, acting with the rhomboid muscles dorsally, help to keep the forelimbs attached to the sternum and chest while preventing them from splaying.

The shoulder joints of cursorial mammals move almost entirely in the sagittal plane while flexing and extending in synchrony with the other joints to help the animal in ground progression. The head of the humerus is larger than the glenoid cavity, making the shoulder joint appear unstable (Sager et al., 2009). Interestingly, shoulder dislocation in animals is rare despite this inherent instability and it occurs much less frequently than dislocation of the apparently more anatomically stable hip joint. The shoulder joint capsule with its glenohumeral ligaments contributes to the joint's stability, although these ligaments are weak and cannot account entirely for maintaining joint movement in the sagittal plane. The juxtaposition of the infraspinatus and subscapularis muscles laterally and medially to the joint contributes considerably to its stability. Both muscles have short, robust tendons which merge with the shoulder joint capsule to stabilise the joint.

The infraspinatus and the subscapularis muscles are often described as facilitating abduction and adduction of the shoulder joint, respectively. However, their role in these animals is rather to prevent adduction and abduction. The equine scapular spine has shifted cranially on the scapula, leaving a relatively larger infraspinous fossa and giving the infraspinatus muscle a more lateral position. The cranial displacement of the equine scapular spine also results in a more cranial positioning of the supraspinatus muscle, facilitating its role as the primary extensor of the shoulder joint (Payne et al., 2004). While the infraspinatus and subscapularis muscles stabilise the shoulder joint from lateral and medial movements, the tendon of *biceps brachii* and a large belly of the long head of the *triceps brachii* also restrict any cranial and caudal shifts of the shoulder joint, respectively.

**FIGURE 10:**
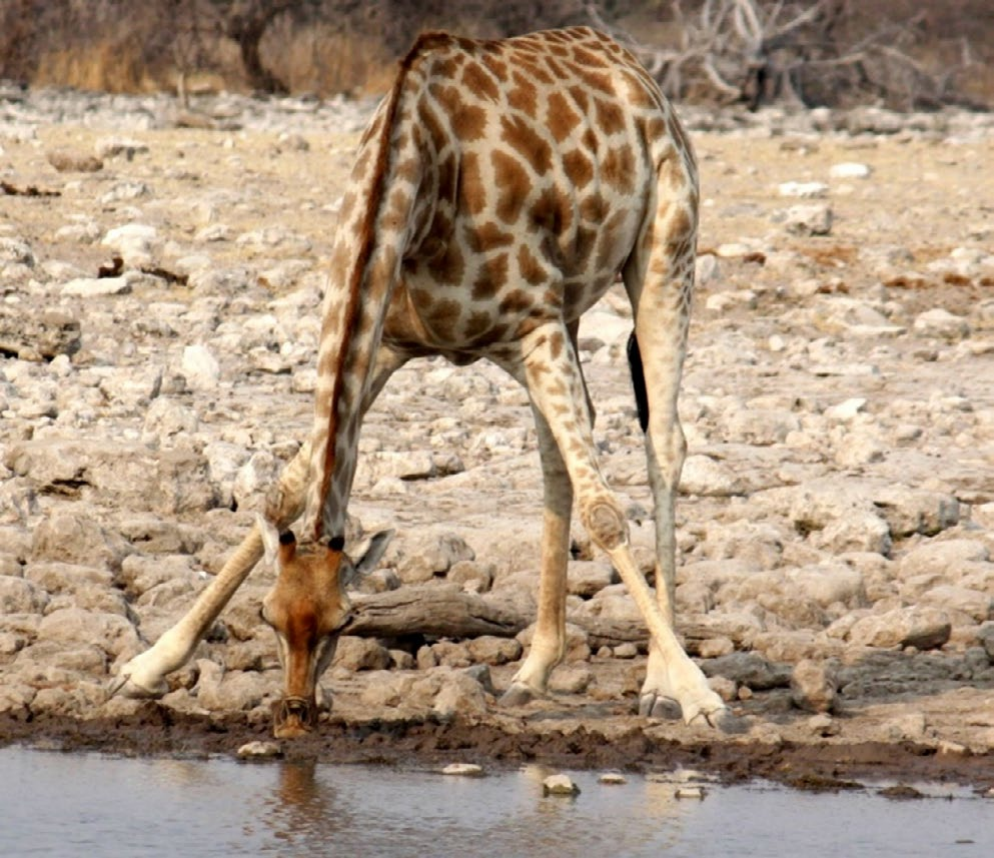
Forelimb abduction by a giraffe drinking water. The image was taken in Namibia and reproduced from the personal collection of Dr Jonathan Holmes

It is interesting to note that the canine triceps brachii has four rather than three heads of origin, and the name *quadriceps* would have been more apt for the muscle. However, such misnomers are common in veterinary anatomy as many terms were adopted from human anatomy. The collar of muscles surrounding the shoulder joints in cursorially specialised quadrupeds virtually transforms its movements into a hinge rather than a ball‐and‐socket joint. However, in some instances, minor abduction and adduction of the forelimbs can occur, although it is not clear whether these movements occur at the shoulder joint or the trunco‐brachial junction. Although such examples are rare, the giraffe, for example, abducts its forelimbs while bringing its neck down to drink water (Figure 10).

Knowledge of vertebrate shoulder joint anatomy is important, because it can provide interesting insights into the human shoulder joint. For example, canine shoulder joints have been used to understand aspects of the human shoulder joint, including its stability mechanisms comprising the glenoid labrum and attachments of the joint capsule and lateral glenohumeral ligament; the complex biomechanics of human shoulder joint, the clinical implications of joint dislocation (Sager et al., 2009), and the anatomy of the rotator cuff muscles (Mathewson et al., 2014). The human shoulder joint is inherently unstable and damage to the rotator cuff muscles due to ageing or trauma is well known. Shoulder pain due to such damage is one of the commonest clinical conditions related to the upper limb in the UK (Bury & Littlewood, 2018).

## 4 INTEGRATION OF COMPARATIVE ANATOMY AND ONE HEALTH IN ANATOMY EDUCATION

To date, the integration of veterinary anatomy and human anatomy curricula has been sporadic, although more and more universities in the UK and USA are offering undergraduate courses in comparative anatomy that are accessible to medical students. Moreover, multiple universities (e.g., University of Edinburgh in the UK, University of Cork in Ireland) are now offering a MSc in (clinical) anatomy with options for studying comparative anatomy. Therefore, opportunities for integrating comparative anatomy into human medicine education are increasing, but further encouragement is needed. In contrast, the veterinary medicine curriculum has embraced the One Health concept more, often integrating knowledge and concepts from human anatomy, physiology and immunology. Furthermore, educational organisations, such as the Council of Education in Public Health and accreditation agencies, such as the Association of American Veterinary Medical Colleges, Association of American Medical Colleges and the Liaison Committee on Medical Education are encouraging convergence between human and veterinary medicine, including anatomy (Stroud et al., 2016). Perhaps one of the most important barriers to the successful integration of the One Health concept into human medicine curricula is the already workload‐heavy curriculum followed in human medical schools, which leaves little or no room for exploration of One Health concepts. Preparing students for medical board certification exams and teaching to the test leaves little room for introducing veterinary knowledge or relevant facets of comparative anatomy.

An effective way to introduce veterinary concepts into the human medical curriculum could be through the elective courses/modules offered by many medical schools. Anatomy can be a bridging subject between these human and veterinary curricula because examples from the animal world, as outlined above, can provide context and a broader vision for human medicine graduates. Communication between the graduates from both human and veterinary medical schools is important, and one of the ways to stimulate communication would be to introduce common learning and research‐based programmes. For example, in University College Dublin, the human medicine school organises the Student Summer Research Awards, during which both human and veterinary medicine students conduct short research projects under the supervision of faculty members from the other discipline. Such research projects can be an excellent way to gain insights into comparative anatomy. Fortunately, many western medical schools maintain strong research profiles with groups engaged comparative and evolutionary biology research, and could also facilitate this.

It is time that more opportunities are made available to medical students to develop a robust understanding of comparative anatomy, physiology and pathology. Informed curriculum mapping (Al‐Eyd et al., 2018) must also identify feasible options for convergence in the medical curriculum. Key One Health concepts will first need to be identified before they can be mapped to curricula to expedite their integration. For example, understanding environmental influences, including zoonotic, vector‐borne and allergen‐related diseases, and building a comprehensible pathophysiologic disease model should be included as primary skills (De Giusti et al., 2019). Similarly, knowledge of zoonotic diseases and a view of human diseases through a lens of the human–animal–environment triad, with a sound grip of evolutionary principles, should be part of the core competencies of medical graduates.

Problem‐solving sessions or case studies may be an effective way of introducing One Health concepts into the medical curriculum. Facilitating student groups and societies to promote One Health concepts to their peers is crucial. An alliance between students of human and veterinary medicine and public health professionals, the International Student One Health Alliance, has already emerged and is active in promoting One Health. Open days in human and veterinary medicine schools may allow medical students and staff to interact with their veterinary counterparts and develop collaborative projects. It will be easiest to introduce such initiatives in universities with both human and veterinary medical schools. In the USA, Tufts University organises a *Comparative Anatomy Exchange Day* to promote interaction between students of human and veterinary medicine (School of Medicine and Cummings School of Veterinary Medicine). A similar exchange event, *the Penn Inter*‐*Health School Anatomy Exchange*, is also arranged by the Perelman School of Medicine and School of Veterinary Medicine of Pennsylvania University. It is important to collect feedback from the students to assess the value of such initiatives and improve such exchanges. A five‐point Likert scale‐based assessment of the effectiveness of the various ways of integrating One Health concepts into medical education based on a carefully designed questionnaire would be one way of beginning to do this. Student organisations should be encouraged to engage with the importance of One Health through discussion sessions and setting up focus groups or student societies.

Events such as medical and veterinary student exchanges provide exciting platforms for collaboration and sharing experiences or perspectives of healthcare. It is important to focus on understanding diseases from the perspective of the interrelationships and shared space between humans, animals and the environment while prioritising ways to exploit the overlapping domains between human and veterinary medicine curricula and incentivise interactions between students and graduates of both disciplines at multiple levels. It will help medical and veterinary professionals comprehend the complexity, multidimensionality and core concepts that define a disease state. Moreover, it will reduce the tendency to work in silos and stimulate collaboration by providing a fertile ground to analyse the astounding diversity of life, who we are, where we come from, our relationship with the animal kingdom and environment; and most importantly, how human activity impacts on environment and human health.

## CONFLICT OF INTERESTS

The authors declare that they have no competing interests.

## Data Availability

The data that support the findings of this study are available from the corresponding author upon reasonable request.
